# *SCN1A* channelopathies: Navigating from genotype to neural circuit dysfunction

**DOI:** 10.3389/fneur.2023.1173460

**Published:** 2023-04-17

**Authors:** Alexander Bryson, Steven Petrou

**Affiliations:** ^1^Ion Channels and Disease Group, The Florey Institute of Neuroscience and Mental Health, The University of Melbourne, Parkville, VIC, Australia; ^2^Praxis Precision Medicines, Inc., Cambridge, MA, United States

**Keywords:** epilepsy, Na_v_1.1, *SCN1A*, neural circuits, homeostatic adaptation

## Abstract

The *SCN1A* gene is strongly associated with epilepsy and plays a central role for supporting cortical excitation-inhibition balance through the expression of Na_V_1.1 within inhibitory interneurons. The phenotype of *SCN1A* disorders has been conceptualized as driven primarily by impaired interneuron function that predisposes to disinhibition and cortical hyperexcitability. However, recent studies have identified *SCN1A* gain-of-function variants associated with epilepsy, and the presence of cellular and synaptic changes in mouse models that point toward homeostatic adaptations and complex network remodeling. These findings highlight the need to understand microcircuit-scale dysfunction in *SCN1A* disorders to contextualize genetic and cellular disease mechanisms. Targeting the restoration of microcircuit properties may be a fruitful strategy for the development of novel therapies.

## Introduction

*SCN1A* encodes the alpha subunit of the voltage-gated sodium channel, Na_V_1.1, and is the gene most strongly associated with epilepsy, implicated in both rare monogenic syndromes and common forms of epilepsy with complex inheritance (1). Since Na_V_1.1 is primarily expressed within inhibitory interneurons and many epilepsy-associated *SCN1A* variants were found to predispose to a loss of channel function, the pathophysiology of *SCN1A* disorders has been conceptualized as driven by an interneuronopathy that predisposes to cortical hyper-excitability through disinhibition (2, 3). However, although disinhibition may precipitate neural circuit dysfunction, there is increasing evidence for widespread alterations to cellular and synaptic properties in mouse models of *SCN1A* disorders, suggesting complex network remodeling that accumulates over development contributing to the disease phenotype (4–6). These findings may be of particular importance given the recent discovery of epilepsy-causing *SCN1A* gain-of-function variants (7–10). Furthermore, the clinical phenotype of SCN1A disorders has expanded to include hemiplegic migraine which possesses a quite distinct pathophysiological basis to epilepsy, suggesting diverse pathways to neural circuit dysfunction (11).

Here, we provide a brief overview of the genetic basis and clinical features of *SCN1A* channelopathies, and then consider their impact upon neuronal and neural circuit function. Although our understanding of the genetic and molecular basis of these disorders has progressed considerably, unraveling neural circuit pathology represents the key challenge for future efforts bridging genotype to phenotype, and may provide important insight for the development of novel therapies that restore network function.

## *SCN1A* channelopathies: Genotype and phenotype

The *SCN1A* gene is located on chromosome 2q24 and contains 26 coding exons, although several splice variants exist which may contribute to differential Na_V_1.1 expression observed during early development (12). For example, the inclusion of the neonatal poison exon 20 (20 N) leads to protein truncation due to a frameshift that incorporates a stop codon, and downregulation of 20 N corresponds with increased post-natal Na_V_1.1 expression (13, 14). *SCN1A* encodes four homologous domains (D1–D4) with each consisting of six transmembrane alpha helical segments (S1–S6). Segments S1–S4 comprise the channel voltage-sensing element, S5 and S6 the pore forming region and ion selectivity filter, and an intracellular loop that sits between domains D3 and D4 acts as an inactivation gate by folding across the channel pore (15). Membrane depolarization causes a rotation and outward movement of segment S4 that induces a conformational change in the pore and channel opening. Intracellular loops between domains D1 and D2, and D2 and D3, contain residues subject to phosphorylation that modify gating properties and a binding site for ankyrin, respectively (16). The latter co-ordinates channel localization at the axon initial segment which is crucial for the functional role of Na_V_1.1 in action potential generation (17).

### Dravet syndrome and GEFS +

*SCN1A* was first associated with epilepsy when a missense mutation was identified in affected members of two families possessing a strong lineage of generalized convulsions and febrile seizures consistent with the clinical syndrome of Genetic Epilepsy with Febrile Seizure Plus (GEFS+) (18). Due to the notable temperature sensitivity observed in patients with GEFS+, a subsequent study in 2001 screened seven patients with a severe developmental and epileptic encephalopathy (DEE) known as Dravet Syndrome, or Severe Myoclonic Epilepsy of Infancy, which is characterized by fever-related seizures and status epilepticus (19, 20). Remarkably, this study identified *SCN1A* variants in all subjects, establishing Dravet Syndrome as the prototypical *SCN1A* channelopathy. Over 80% of cases of Dravet Syndrome (DS) are caused by *SCN1A* variants, and 80–90% of these arise through *de novo* mutations. DS presents within the first year of life with recurrent generalized and focal clonic (often hemi-clonic) convulsions frequently triggered by fever (21). Status epilepticus is common and, over subsequent years, myoclonus, focal impaired awareness, atonic, and atypical absence seizures can emerge. Although development may be normal or mildly impaired at diagnosis, nearly all patients will experience developmental delay culminating in an often severe intellectual disability, ataxia, and motor impairment (22). Seizures are refractory to pharmacotherapy and antiseizure medications that act through sodium channel blockade are contraindicated as they may aggravate seizures and worsen developmental outcomes.

More than 1,200 *SCN1A* variants have been associated with DS, and over 50% of these cause protein truncations through nonsense, frameshift, or splice site mutations, suggesting that Na_V_1.1 haploinsufficiency drives the clinical phenotype. Interestingly, DS-causing variants within the non-coding site of intron 20 were recently identified which promote the inclusion of exon 20 N and downregulation of functional Na_V_1.1 (13). Missense mutations within coding regions that alter channel function tend to cluster within pore-forming segments S5 and S6 and although the functional impact of these variants can be difficult to predict based on their coding sequence alone, a review of patch-clamp data across 26 missense DS cases found that 20 exhibited a reduction of channel conductance consistent with a loss-of-function (23). The remaining six exerted mixed effects on conductance and gating properties that, when considering ion channel biophysics and neuron excitability, could potentially exert either gain or loss-of-function.

**Figure 1:**
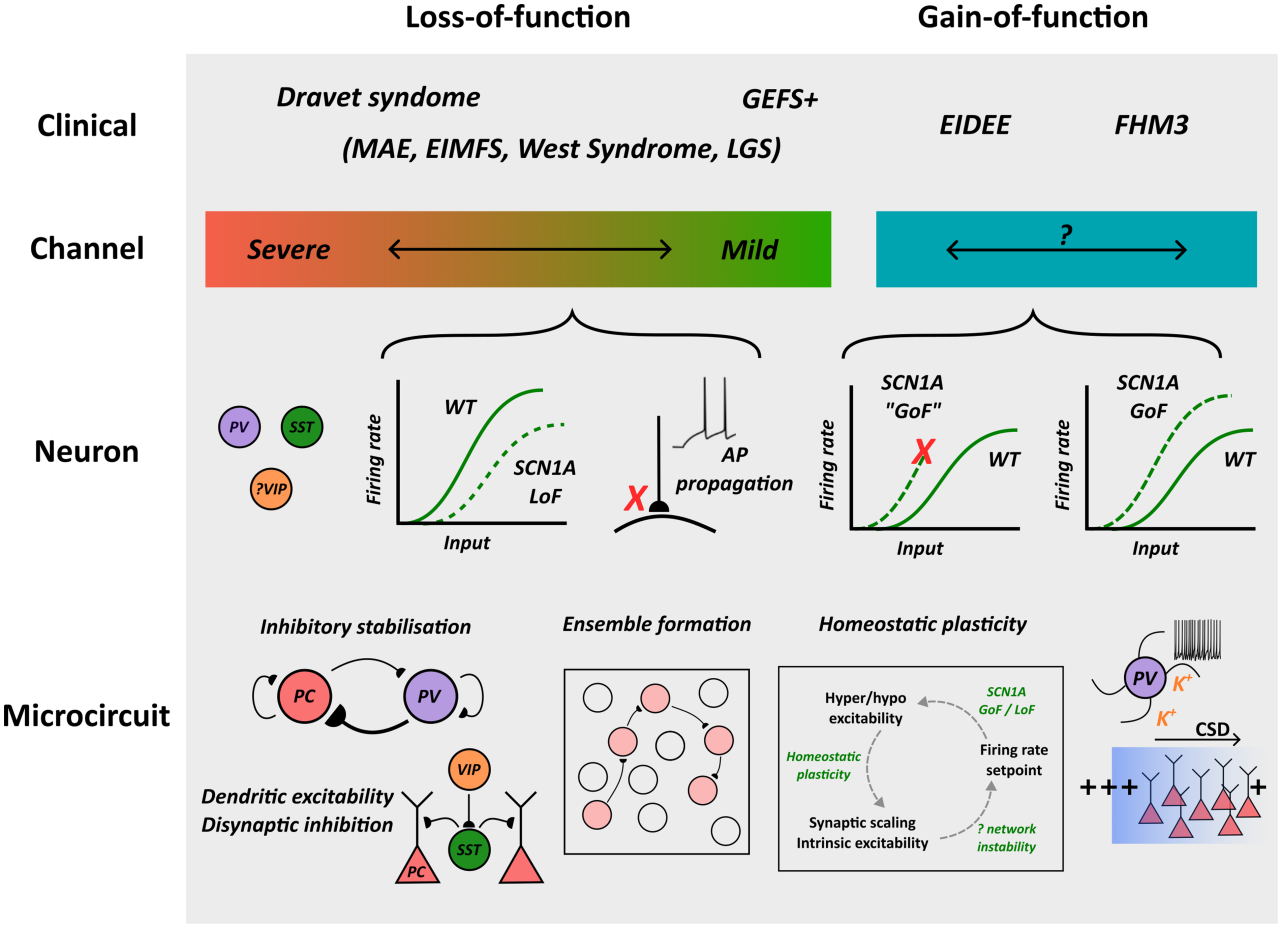
Overview of pathological mechanisms implicated in *SCN1A* channelopathies. Na_V_1.1 loss and gain-of-function are associated with distinct clinical syndromes, and whereas profound loss-of-function is associated with Dravet Syndrome, an association between the extent of channel gain-of-function and clinical phenotype has been proposed but not confirmed. Loss-of-function can lead to interneuron hypo-excitability or impaired action potential propagation/transmission, and gain-of-function may enhance excitability or predispose to neuronal depolarization block. Although microcircuit-scale pathology remains to be fully elucidated, possible mechanisms contributing to cortical dysfunction are outlined. MAE: myoclonic astatic epilepsy; EIMFS: epilepsy of infancy with migrating focal seizures; LGS: Lennox–Gastaut syndrome; GoF: gain-of-function; LoF: loss-of-function; AP: action potential.

GEFS + is a familial epilepsy syndrome characterized by the presence of febrile seizures, febrile seizures plus—a term used to describe febrile seizures beyond 6 years of age or coexisting afebrile generalized tonic–clonic seizures—and other generalized or focal seizure types (24). Between 10 and 20% of families with GEFS+ harbor an *SCN1A* mutation, and although autosomal dominant inheritance with variable penetrance is observed in most cases, *de novo* mutations and cases with recessive inheritance can occur (25). All *SCN1A* variants associated with GEFS + have been found to comprise missense mutations that produce either channel loss-of-function or potentially mixed biophysical effects (26–28). An *in silico* pathogenicity prediction tool did not predict a difference in severity of channel dysfunction between DS versus GEFS + missense variants, although GEFS + variants were less likely to produce a loss of Na_V_1.1 conductance which may suggest a spectrum of channel dysfunction that correlates with clinical severity (Figure 1) (23, 29). An important caveat is the presence of variants that disrupt Na_V_1.1 post-translational modification and membrane expression *in vivo* (30, 31). Here, functional studies may either over or underestimate channel dysfunction depending upon the experimental conditions used.

### Other epilepsy syndromes and familial hemiplegic migraine

*SCN1A* variants have recently been implicated in a form of DEE considered more severe than DS, known as early infantile developmental and epileptic encephalopathy (EIDEE). In 2017, nine patients were identified with seizure onset within 3 months of age, developmental delay within 6 months and a hyperkinetic movement disorder (32). Patients developed a severe intellectual disability with profound language impairment, were non-ambulant, and all possessed *SCN1A* missense variants. Remarkably, eight harbored the T226M variant in exon 5 located in segment S4 of domain D1. The clinical spectrum of *SCN1A* EIDEE has since expanded to include a syndrome of neonatal epilepsy with movement disorder and arthrogryposis multiplex congenita—a condition characterized by contractures involving multiple body regions related to reductions of fetal movement (7, 33). Patients with this condition also develop a hyperkinetic movement disorder, experience multiple seizure types, are non-verbal and non-ambulant, and *SCN1A* missense variants have been identified in all, with clustering observed at segments S4 and S5 and the intracellular linker between D3 and D4 that regulate voltage sensing and channel inactivation (7). Interestingly, these regions are implicated in gains of channel function, and biophysical characterization of several variants have revealed impaired channel inactivation and enhanced current (7, 9). Rarely, *SCN1A* variants are implicated in other forms of DEE including myoclonic astatic epilepsy, epilepsy of infancy with migrating focal seizures, West Syndrome and Lennox–Gastaut Syndrome (21, 34, 35). In contrast to disorders caused by single gene mutations, ultra-rare SCN1A variants are thought to heighten the risk of common epilepsies associated with polygenic inheritance. Indeed, genome-wide association studies have identified an association between single-nucleotide polymorphisms of *SCN1A* and both genetic generalized epilepsy (GGE), and mesial temporal lobe epilepsy with hippocampal sclerosis and a history of febrile seizures (36, 37). Furthermore, whole exome sequencing performed in patients with GGE and focal epilepsy revealed that *SCN1A* was in the top 10 genes enriched in both cohorts (1).

The phenotype of *SCN1A* channelopathies has expanded beyond epilepsy to include a rare form of hemiplegic migraine often inherited in autosomal dominant fashion with incomplete penetrance, known as familial hemiplegic migraine (FHM) (11). FHM is associated with missense variants of *SCN1A* (FHM3), *CACNA1A* which encodes the P/Q-type voltage-dependent calcium channel (FHM1), and *ATP1A2* which encodes a sodium-potassium ATPase (FHM2) (38). Sporadic hemiplegic migraine caused by *de novo SCN1A* mutations have also been observed. FHM presents with recurrent migraine auras manifesting as hemiplegia, aphasia, visual field deficits or, in the case of FHM1 and FHM2, even impaired consciousness and coma (38, 39). There are several reports of FHM3 co-occurring with epilepsy, although in these cases clinical attacks occur independently and the nature of the pathophysiological overlap is unclear (40). FHM3 *SCN1A* variants tend to cluster between domains D3 and D4 and, similar to SCN1A-related EIDEE variants, produce either mixed or gain-of-function channel effects including enhanced persistent current related to incomplete channel inactivation (7). Indeed, it has been argued that the gain-of-function found in FHM3 is even more pronounced than EIDEE *SCN1A* variants (7).

## *SCN1A* channelopathies: Channel biophysics, neuronal excitability, and regional effects

Although *SCN1A* is firmly established as a gene that is crucial to the pathophysiology of epilepsy, the relationship between genotype and phenotype is complex and at times counterintuitive. Therefore, to untangle these issues considerable effort has been made to characterize the impact of disease-causing variants on both Na_V_1.1 channel and neuron function (41). Here, we will consider the relationship between channel dysfunction in *SCN1A* variants and neuron excitability, with an emphasis upon studies characterizing neuronal properties using *in vivo* and *ex vivo* disease models.

### Na_V_1.1 biophysics, neuronal excitability, and loss-of-function

Na_V_1.1 is expressed within inhibitory interneurons and preferentially distributed within the axon initial segment, nodes of Ranvier, and soma to confer an important role for action potential initiation and propagation (2, 17). Na_V_1.1 is strongly expressed within parvalbumin (PV)-positive interneurons, but also found in somatostatin (SST) and vasoactive intestinal peptide-expressing (VIP) interneurons. Although Na_V_1.1 has been detected in excitatory pyramidal cells its functional significance in this neuronal type is uncertain. Na_V_1.1 possesses a more depolarized voltage of half-activation and inactivation compared to other voltage-gated sodium channels, such as Na_V_1.6, and computational modeling suggests that this property confers an ability to sustain high-frequency firing rates within PV interneurons that may help to maintain excitation-inhibition balance (9).

Most DS causing mutations produce protein truncation generating non-functional channels, and the majority of missense variants result in complete or partial loss of channel conductance due to amino acid substitutions involving the pore-forming region or inactivation gate (42, 43). Therefore, as a proxy for understanding the impact of these changes upon neuronal function, mice heterozygous for an *Scn1a* null allele (*Scn1a*^+/−^) have been developed that recapitulate core features of DS including spontaneous and thermal seizure susceptibility and behavioral changes (2, 44, 45). Consistent with the expression profile of *Scn1a*, whole cell patch clamp recordings from inhibitory interneurons in brain slices of *Scn1a*^+/−^ mice have consistently shown reductions in sodium current density and multiple electrophysiological features of impaired intrinsic excitability (46–48). Both PV and SST interneurons exhibit increased rheobase and a more depolarized action potential threshold compared to wild-type, likely due to preferential expression of Na_V_1.1 within the axon initial segment, and reductions of peak firing rate and neuronal gain (Figure 1) (5, 46). Alterations in the characteristics of action potential trains have also been observed, including greater failure rates during high frequency firing and “stuttering” electrophysiological characteristics in PV interneurons (5, 48). These changes to intrinsic interneuron properties appear to be developmentally regulated. A recent study assessed PV interneuron function in somatosensory cortex using a technique to obtain slice recordings at both early (P16-22) and late (> P35) timepoints in wild-type and *Scn1a*^+/−^ mice (48). This revealed that intrinsic excitability had normalized with maturation, presumably due to compensatory upregulation of other Na_V_ subtypes, yet a severe epileptic phenotype persisted suggesting another pathological locus. Interestingly, paired recordings from neighboring pyramidal neurons and targeted optogenetic stimulation of PV interneurons revealed a selective deficit in the fidelity of action potential propagation and synaptic transmission, suggesting that a major contributor of Na_V_1.1 loss of conductance to the pathophysiology of DS could relate to its role at the nodes of Ranvier (Figure 1) (48).

The role of VIP interneurons to the pathogenesis of DS has been less extensively studied. VIP interneurons exert a unique role in cortical function through disinhibiting other interneuron subtypes, particularly SST interneurons (49). Recently, it was found that the excitability of a subpopulation of VIP interneurons that exhibit irregular spiking characteristics appear preferentially disrupted in *Scn1a*^+/−^ mice, with increased action potential threshold and reduced peak firing rates (50). Given these changes would be expected to enhance the inhibitory influence of SST interneurons, the relevance for the emergence of seizures is unclear. Nevertheless, irregular spiking VIP interneurons are modulated through cholinergic inputs and may help co-ordinate learning by regulating synaptic plasticity, and so these results raise interesting questions regarding the cognitive impairment associated with DS^51^.

In contrast to the well-established inhibitory interneuron dysfunction found in *Scn1a*^+/−^ mice, the involvement of excitatory pyramidal cells is less defined, and mice with recombinase-dependent heterozygous *Scn1a* deletion in pyramidal cells do not exhibit a discernible phenotype (51). However, co-deletion of *Scn1a* in both pyramidal cells and inhibitory interneurons improved seizure severity and lethality compared to mice with deletion within inhibitory interneurons alone, suggesting that loss of Na_V_1.1 in pyramidal cells exerts an ameliorating effect (51). Unexpectedly, strain-selective increases of sodium current have been observed in *Scn1a*^+/−^ mice, and studies assessing pyramidal cell excitability across development have revealed reductions of intrinsic excitability during periods of severe disease, although it is unclear to what extent these findings are due to homeostatic adaptations (4, 52). Nevertheless, most studies have demonstrated unaltered pyramidal cell excitability and, overall, Na_V_1.1 loss-of-function appears to exert broad deficits of inhibition by impairing multiple aspects of intrinsic excitability across different interneuron populations (46, 53).

Apart from a simple loss of conductance, DS and GEFS + *SCN1A* missense variants have also been shown to disrupt a range of other channel properties such as the voltage-dependence and kinetics of gating (41, 43). For some variants, the impact of these functional alterations on neuronal excitability can be difficult to predict. For example, the R1648H variant associated with GEFS + was found in heterologous expression systems to enhance the magnitude of persistent sodium current and broaden the “window current” associated with fast activation and inactivation, consistent with a gain-of-function, yet it impaired recovery from slow inactivation suggestive of a loss-of-function (27, 28). To disentangle these effects upon neuronal excitability, a heterozygous knock-in R1648H mouse model (*Scn1a*^RH/+^) was developed (54). Although sodium current densities were similar, both hippocampal and cortical fast-spiking inhibitory interneurons had lower peak firing rates in *Scn1a*^RH/+^ mice compared to wild-type. Spike threshold was unchanged between genotypes, but a possible deficit in action potential initiation was revealed through phase plot analysis, perhaps due to delayed recovery from slow inactivation, and further suggesting that axon initial segment dysfunction may be an important aspect of cellular pathology in DS^55^.

### Na_V_1.1 gain-of-function

Recently, the biophysical characterization of severe EIDEE *SCN1A* variants has shifted the view that loss of Na_V_1.1 channel function is required for an epileptic phenotype. Analysis of the T226M variant found changes consistent with channel gain-of-function, including hyperpolarizing shifts of fast activation that increase window current and potentially lowering action potential threshold, and a faster recovery from inactivation to enhance channel availability (9). A subsequent analysis of three further EIDEE variants also revealed faster recovery from inactivation and depolarizing shifts of inactivation, supporting these first observations (7). Using dynamic action potential clamp and detailed single neuron modeling it was found that although these changes may enhance the magnitude of sodium current they can also predispose to neuronal depolarization block at higher firing frequencies (Figure 1), emphasizing the need to consider broader neuronal dynamics when drawing conclusions about the impact of channel dysfunction (9). However, these findings require confirmation in an animal model, and it is unclear if similar findings hold for other EIDEE associated variants.

Interestingly, analysis of FHM3-associated *SCN1A* variants has also consistently revealed changes to support Na_V_1.1 gain-of-function, including enhanced recovery from fast and slow inactivation and increased persistent sodium current (55–57). Indeed, increased persistent current, in particular, appears to be a more prevalent feature of FHM3 compared to EIDEE associated *SCN1A* variants (7). Furthermore, the impact of these changes on neuronal excitability has also been clarified by the development of an *Scn1a* L1649Q knock-in mouse model of FHM3 (8). Here, nucleated patch recordings confirmed the presence of enhanced persistent sodium current inferred from results in expression systems, and fast spiking interneurons in both cortex and hippocampus were found to be hyperexcitable compared to wild-type, exhibiting increased gain, higher peak firing rates, and generating a greater frequency of spontaneous inhibitory post-synaptic currents onto excitatory pyramidal cells (Figure 1). Pyramidal cell excitability, in contrast, was unaffected, suggesting that enhanced inhibitory activity plays an important role in FHM3 pathophysiology (8).

### Interneuron subtypes and regional determinants of phenotype

The contribution of distinct brain regions and cell types to the clinical phenotype of *SCN1A* disorders has been examined through Cre-recombinase dependent gene deletion. Heterozygous deletion of *Scn1a* in either PV or SST interneurons was found to be sufficient to induce thermal seizure susceptibility in mice, although selective deletion within PV interneurons was associated with a more severe phenotype and deletion in both populations exerted a synergistic impact with respect to both seizure susceptibility and duration (53). Surprisingly, it was found that behavioral changes may associate with dysfunction of distinct cell types: PV *Scn1a* deletion led to autistic-like traits, whereas SST deletion predisposed to a hyperactive phenotype (53).

Regional *Scn1a* deletion has helped delineate the role of different brain structures to disease features. Targeted deletion within forebrain inhibitory interneurons, achieved with selective expression of Cre-recombinase under control of the *dlx1/2* enhancer, could recapitulate most features of global *Scn1a*^+/−^ knockout mice including thermally induced and spontaneous seizures that lead to premature death, deficits in spatial learning, and hyperactive and autistic-like behavior (44, 45). Interestingly, social and memory deficits could be rescued with GABA_A_ receptor positive allosteric modulators suggesting that the fundamental integrity of neural circuits in *these* mice remain intact (45). Forebrain Na_V_1.1 deletion could also account for abnormalities in sleep architecture observed in DS, such as reduced delta frequency power and sleep spindle frequency; however, circadian abnormalities were only observed in *Scn1a*^+/−^ mice perhaps due to involvement of the suprachiasmatic nucleus (58). Further insight into seizure pathophysiology has been achieved using viral injection of Cre-recombinase into *Scn1a Floxed* mice enabling localized gene knockout. Both cortical and hippocampal *Scn1a* deletion predisposed to hyperthermic, focal, and generalized seizures, although interestingly seizure frequency had improved by P21 suggesting compensatory adaptations that can suppress a hyperexcitable phenotype (59, 60). Focal hyperexcitability within hippocampal circuits of *Scn1a*^+/−^ mice has also been demonstrated in response to pharmacological and electrical stimulation, supporting the importance of hippocampal involvement in the pathophysiology of DS (61, 62).

### Limitations

Exploration of Na_V_1.1 biophysics and neuronal dysfunction has provided inroads into the pathophysiology of *SCN1A* channelopathies by revealing deficits of excitability across several interneuron populations, a correlation between profound loss-of-function and the more severe phenotype of DS, and an interesting divergence between Na_V_1.1 gain-of-function and the distinct clinical syndromes of EIDEE and FHM3. Yet several outstanding questions remain. For example, some DS *SCN1A* variants exert seemingly mild Na_V_1.1 channel loss-of-function or even mixed biophysical effects, yet it remains unclear why severe clinical consequences can arise (42, 54). Furthermore, the association between EIDEE and *SCN1A* gain-of-function points toward alternative mechanisms for hyperexcitability in SCN1A channelopathies, and while gain-of-function is observed in both FHM3 and EIDEE their clinical consequences differ dramatically. One limitation is that techniques to characterize intrinsic excitability incompletely capture the impact of channel biophysics on neuronal function. For instance, alterations such as enhanced persistent sodium current may modulate subthreshold properties including synaptic integration and resonance which are not explored with standard electrophysiological protocols. Furthermore, neurons *in vivo* are bombarded with synaptic inputs that fluctuate over rapid timescales, and changes to Na_V_1.1 properties may exert distinct effect on neuronal properties under this environment (63). For these reasons, combined with the continued development of novel techniques enabling microcircuit-scale population recordings, there has been a recent shift in focus toward understanding the basis of network dysfunction in *SCN1A* disorders.

## *SCN1A* channelopathies and microcircuit dysfunction

Many of the insights derived from molecular-genetic studies of *SCN1A* channelopathies remain conceptually distinct from the defining pathophysiology of epilepsy: a neuronal network predisposed to generating bursts of synchronous or hyper-excitable activity. Clarifying the interaction between these descriptions is of utmost importance for both a deeper understanding of disease pathogenesis and therapeutics (64). For example, while strategies to enhance inhibitory interneuron activity may seem a logical treatment approach at face value, the role of interneuron dysfunction in seizures is highly complex and governed by the network connectivity and dynamics in which they are embedded (65). Second, intrinsic neuron and synaptic properties may be modified through secondary homeostatic compensations that aim to restore favorable network properties, and it is possible these processes may lead to unpredictable effects upon cortical excitability in genetic channelopathies (66).

### Interneurons and ictal network activity

Beyond *SCN1A* disorders, an extensive number of studies have shown that interneurons are closely associated with the generation of interictal and ictal network discharges, yet their role is complex and appears dependent upon cell type, the underlying epilepsy model, and the spatiotemporal relationship with seizure onset. For example, optogenetic activation of PV interneurons can enhance neuronal synchrony within the seizure-onset zone in a 4-aminopyridine (4-AP) chemo-convulsant seizure model, yet distal activation can suppress ictal propagation and duration (67). A differential influence of PV interneurons has also been observed with respect to the timing of seizure onset. In a pilocarpine model optogenetic stimulation of PV interneurons switched from exerting an anti-seizure to pro-seizure influence within seconds of ictal onset, and anti-seizure effects could be restored through KCC2 overexpression, suggesting that collapse of the pyramidal cell chloride gradient and GABA reversal potential may contribute to the excitatory impact of PV stimulation (68).

Parvalbumin interneurons are also implicated in the generation of inter-ictal epileptiform discharges (IEDs), although, again, it remains unclear whether such involvement represents an inhibitory “restraint” upon excitatory activity or instead a trigger that could potentially initiate ictal onset (69). In a pilocarpine model of TLE, axo-axonic PV interneurons precede pyramidal cell recruitment during IEDs and the selective activation of PV interneurons can induce IED-like discharges in the presence of 4-AP (70, 71). However, in support of a suppressive role, 2-photon calcium imaging performed in cortical layer 2/3 after 4-AP administration revealed matched recruitment of pyramidal cells and PV interneurons during IEDs, but a deficit of PV recruitment at seizure onset, suggesting that breakdown of PV inhibition may predispose to unrestrained activity (72). SST interneurons have generally been shown to prevent ictal activity, and optogenetic stimulation can suppress seizures under different experimental paradigms (65). The anti-ictal influence of SST interneurons may arise through suppression of dendritic excitability, spatial restriction of excitatory activity through di-synaptic inhibition, and their lower propensity to indue oscillatory network activity (Figure 1) (49).

Although imaging spontaneous seizures in *Scn1a*^+/−^ mice is difficult due to their infrequent occurrence, several recent studies have applied similar techniques to explore peri-ictal interneuron dynamics during thermal-induced seizures (73). Unexpectedly, higher baseline PV firing rates were found in *Scn1a*^+/−^ mice and, prior to seizure onset, desynchrony within the PV population and between PV and PC neurons was observed. These changes did not occur with increased temperature in wild-type mice, perhaps reflecting a failure of PV-generated feedback inhibition during heightened activity as a driver of seizures in *Scn1a*^+/−^ mice (73). Differential recruitment of interneuron subtypes during the pre-ictal period has also been observed. Interestingly, compared to PV and VIP interneurons, SST interneurons were recruited much earlier, with a significant lag of ~ 3 s prior to seizure onset (74). Furthermore, VIP interneurons were sub-maximally activated which may be of relevance given their prominent role for mediating SST disinhibition. Nevertheless, it remains unclear precisely how these findings relate to ictogenesis, and further work is required to dissect the contribution of interneuron subtypes to ictal network activity.

### Interneurons and cortical microcircuit properties

Despite recognition that *SCN1A* disorders impair the excitability of several neuron subpopulations, PV interneurons, in particular, appear to be central mediators of disease pathophysiology: electrophysiological studies have consistently observed deficits within this subtype, selective optogenetic perturbation is closely linked to ictogenesis, and selective *Scn1a* deletion within PV interneurons culminates in a more profound phenotype compared to deletion within other neuron populations (46, 53). These observations are significant given the emerging evidence for the role of PV interneurons for stabilizing excitatory activity in cortical circuits. During early post-natal development in rodents, there is an upregulation of synaptogenesis and a strengthening of recurrent pyramidal cell connections, leading to high excitatory gain in cortical circuits (75). These changes coincide with higher pyramidal cell firing rates, the transition from a discontinuous to continuous pattern on EEG, and the emergence of self-sustained neural activity (76). Cortical networks with high excitatory gain are capable of supporting computational functions such as ensemble formation and pattern completion required for memory storage, but are also susceptible to generating seizures through unrestrained activity unless stabilized by strong recurrent inhibition, known as an inhibition-stabilized network (Figure 1) (77). Interestingly, concurrent with excitatory synaptogenesis is a strengthening of PV-mediated axo-axonic and peri-somatic basket cell inhibition, and enhancement of the transmembrane chloride gradient through KCC2 upregulation to promote more hyperpolarizing GABAergic responses, together suggesting that PV interneurons play a crucial role in ISN formation (78, 79). Furthermore, in a computational model incorporating different interneuron subtypes, it was found that the intrinsic and synaptic features of PV interneurons, in particular their rapid response properties and short synaptic latencies, are particularly suited for conferring stabilizing inhibitory feedback (80). Thus, PV-mediated stabilizing inhibition may represent a “choke point” for seizure restraint, and a facilitator of normal cortical maturation, that is disrupted through *SCN1A* channelopathies (81).

The use of population imaging modalities with single neuron resolution has enabled increasingly fine-grained exploration into cortical microcircuit function and, in turn, how these processes are disrupted during ictogenesis. In healthy cortex, neural activity is characterized by the presence of spatiotemporally co-ordinated patterns of activity, known as ensembles, which are thought to represent fundamental units of brain computation (Figure 1) (82). Ensembles arise within a potentially very high-dimensional space and the ability of cortex to both sustain large numbers of distinct patterns and flexibly switch between ensembles is thought to depend crucially upon intact interneuron function. For example, suppression of PV interneurons limits the functional repertoire and spatial representation of cortical ensembles during visual stimulation (83). Furthermore, subsets of SST interneurons can support task-specific ensemble formation, perhaps through modulating dendritic synaptic plasticity, and which, in turn, are co-ordinated by VIP-mediated SST suppression (84). Importantly, characteristics of neuronal ensembles differ in neuropsychiatric disorders and, in the case of epilepsy, abnormally formed ensembles may represent the microscale substrate from which seizures arise (85, 86). For example, recordings from dentate gyrus slices in a pilocarpine mouse model of TLE revealed that epileptic ensembles were less integrated into the background activity yet more spatially confined, and recruited in a variable manner during interictal-like discharges (87). In a chemo convulsant model of focal epilepsy, the ictal onset zone was characterized by lower dimensional activity, whereby stereotyped bursts of distinct ensembles were recruited in a stepwise fashion prior to seizure propagation (85). Interestingly, PV interneuron recruitment was upregulated before seizure onset, albeit in a spatially heterogeneous manner, suggesting a role for compartmentalizing such “micro-seizure” activity. These findings are of relevance for the mechanisms of both ictogenesis and the associated cognitive deficits observed in *SCN1A* channelopathies, although as yet ensemble dysfunction during spontaneous or acute seizure induction in *Scn1a*^+/−^ mice has not been assessed.

### Homeostatic plasticity

Studies exploring synaptic function and population neural activity in mice have revealed changes that add unexpected complexity to the pathophysiology of *SCN1A* disorders. Surprisingly, cortical spike rates and baseline LFP power spectra were similar between wild-type and *Scn1a*^+/−^ mice under urethane anesthesia, and 2-photon calcium imaging has in fact demonstrated greater PV interneuron activity compared to wild-type (5, 73)—observations that are difficult to reconcile with impaired intrinsic interneuron excitability demonstrated in slice recordings. It is possible these findings may be driven in part by homeostatic adaptations that lead to protective or maladaptive changes that accumulate during development, and potentially predispose to epilepsy (66). Homeostatic plasticity can act to restore physiological network parameters through alterations of intrinsic neuron excitability and the scaling of excitatory and inhibitory synaptic strengths. For example, in temporal lobe epilepsy, compensatory changes in HCN channel expression were observed in excitatory granule cells within the dentate gyrus (88), and downregulation of voltage-gated potassium channels was found to negate and reverse pyramidal cell hypo-excitability due to *SCN2A* loss-of-function in a mouse model (89). Seizure-induced upregulation of RE-1 Silencing Transcription Factor has been identified as a candidate pathway that can modulate both excitatory synaptic strength and voltage-gated sodium channel expression, although its precise role in homeostatic plasticity remains uncertain (66).

While few studies have systematically examined homeostatic plasticity in *SCN1A* disorders there is mounting evidence for compensatory network alterations that are likely to influence seizure susceptibility. Interestingly, in P16-18 *Scn1a*^+/−^ mice, enhanced spontaneous EPSC amplitudes onto pyramidal cells and IPSC amplitudes onto PV interneurons were demonstrated, in addition to reductions of intrinsic PV and SST excitability, although the impact of such complex synaptic changes are difficult to predict at the network scale (5). More intuitively, recovery of PV interneuron function has been demonstrated at later timepoints in *SCN1A*^+/−^ mice, likely due to upregulation of other Na_V_ subtypes, and in a model of the A1783V mutation reductions of intrinsic pyramidal cell excitability during the most severe stages of disease was found, perhaps representing a compensatory response to limit network excitability (4). Using an *in vitro* model of homeostatic plasticity by enhancing network activity with 4-AP, a reduction of pyramidal cell excitability was demonstrated in *Scn1a*^+/−^ mice although this finding varied according to the age of tissue slice (90).

Given the surprising finding that *SCN1A* gain-of-function is associated with severe forms of epilepsy, a modeling study explored the impact upon network behavior of different forms of homeostatic plasticity that act to restore basal pyramidal cell firing rates (Figure 1) (10). Here, it was found that scaling of excitatory and inhibitory synaptic strength, as opposed to changes of intrinsic excitability through altered firing threshold, could preferentially modify network stability and predispose to epileptiform-like activity. These findings offer one explanation for how *SCN1A* gain-of-function mutations unexpectedly predispose to network hyper-excitability, and the recruitment of different homeostatic pathways could account for phenotypic variability observed in epilepsy-causing gene mutations (91). Nevertheless, these hypotheses require experimental validation in an animal model.

### Familial hemiplegic migraine and microcircuit dysfunction

Although some overlap between the pathophysiological basis of epilepsy and migraine has been postulated, cortical network dysfunction observed in migraine has a distinct phenomenology associated with the presence of a spreading wave of neuronal depolarization block, known as cortical spreading depolarization (CSD) (92). CSD is associated with large shifts in transmembrane ionic gradients, particularly an increase in extracellular potassium, cell swelling and has been observed in the context of stroke and traumatic brain injury whereby it may contribute to cellular death. FHM3 mouse models harboring *Scn1a* gain-of-function have demonstrated a propensity for generating CSD and shed light on the pathophysiology of this disorder (8, 93). Remarkably, CSD could be induced through either pharmacological or optogenetic activation of inhibitory interneurons, including the specific activation of PV interneurons alone, and the onset of CSD was accompanied by increased extracellular potassium (93). A proposed unifying mechanism is that interneuron hyperexcitability can produce extracellular accumulation of K^+^, and when the concentration reaches a threshold this predisposes to membrane instability and neuronal depolarization block that can propagate throughout cortex (Figure 1) (93).

## Conclusion and future directions

The last decade has witnessed a tremendous growth in our understanding of the pathophysiology of *SCN1A* channelopathies, due in part to the identification of disease-causing variants with novel biophysical consequences, and the development of mouse models enabling the characterization of neuronal impairment across different cellular populations (44). These advances have also revealed unexpected developmental changes to intrinsic neuron excitability, widespread alterations to synaptic properties, and an epileptic phenotype associated with Na_V_1.1 gain-of-function that suggest the emergent pathology of *SCN1A* disorders can be counterintuitive when the scope of investigation is restricted to the genetic, molecular, or single neuron scale (5, 7, 10, 48).

Unraveling cortical dysfunction in *SCN1A* disorders will require extending our understanding of the role of distinct neuronal populations to microcircuit activity, and across a range of developmental epochs and brain regions. Techniques involving conditional deletion of *Scn1a* within fine-grained cellular populations, precisely interrogating network function with all-optical electrophysiology, and the development of *SCN1A*-associated EIDEE gain-of-function mouse models will be invaluable for tackling these questions (94, 95). Finally, characterizing the brain transcriptomic profile in mouse models across development, and in response to distinct *SCN1A* variants, may help classify homeostatic adaptations unique to different disease phenotypes (66).

These insights will carry therapeutic implications as treatments simply aimed at recovering interneuron excitability may be insufficient to correct the key pathology driving cortical dysfunction. Instead, treatments that correct microcircuit properties such as excitation-inhibition balance, or that are optimized to restore normal dynamic activity between cellular populations may ultimately prove more beneficial for rescuing the clinical phenotype.

## Author contributions

AB and SP wrote and edited the manuscript. All authors contributed to the article and approved the submitted version.

## Funding

This work was supported by a Brain Foundation research grant awarded to AB.

## Conflict of interest

SP is a paid employee and equity holder of Praxis Precision Medicines.

The remaining authors declare that the research was conducted in the absence of any commercial or financial relationships that could be construed as a potential conflict of interest.

## Publisher’s note

All claims expressed in this article are solely those of the authors and do not necessarily represent those of their affiliated organizations, or those of the publisher, the editors and the reviewers. Any product that may be evaluated in this article, or claim that may be made by its manufacturer, is not guaranteed or endorsed by the publisher.
